# Primary mediastinal hydatidosis successfully treated with combined medical therapy and VATS (Video-assisted thoracoscopic surgery)

**DOI:** 10.1016/j.idcr.2020.e01008

**Published:** 2020-11-16

**Authors:** Almurtada Razok, Maisa Mohamed Bashir Ali

**Affiliations:** a Department of Internal Medicine, Hamad Medical Corporation, P.O 3050, Doha, Qatar; b Department of Infectious Diseases, Hamad Medical Corporation, P.O 3050, Doha, Qatar

**Keywords:** Echinococcus, Hydatid cyst, Mediastinum, Video-assisted thoracoscopic surgery, Qatar

## Abstract

Mediastinal involvement of hydatidosis is rare even in endemic areas. Isolated mediastinal without lung or liver involvement is even less commonly reported. We present the case of a young gentleman who was diagnosed with primary mediastinal hydatidosis based on clinical, radiological and pathological criteria. He underwent successful resection of the lesion by VATS (Video-assisted thoracoscopic surgery) preceded by two weeks of medical treatment with albendazole and had an excellent outcome. To the best of our knowledge, this is the first reported case in the state of Qatar.

## Highlights

• Hydatidosis is an endemic infection and can involve a variety of organ systems.
• The mediastinum is one of the rarest locations of hydatidosis.
• It can occur primarily or secondarily to intrathoracic and intraabdominal Echinococcus.
• Surgical treatment remains very crucial in the management of these cases.
• The exact mechanism by which primary mediastinal hydatidosis occurs remains a debatable subject.

## Introduction

Hydatidosis is an endemic infection caused by the larval form of *Echinococcus granulosus.* It is usually acquired through direct contact with an infected dog or by ingestion of food contaminated with eggs. Humans are accidental hosts, with dogs and sheep being the definitive and intermediate hosts, respectively. The most commonly involved organs are the liver and lungs [1], however other organs such as the spleen, kidneys, pancreas and central nervous system can be affected as well. A variety of symptoms can lead to the detection of visceral hydatidosis depending on the involved organ and its nearby structures. Pancreatic head cysts for example can present with jaundice, while body and tail involvement can manifest as abdominal pain and vomiting [2].

## Case report

**Fig. 1:**
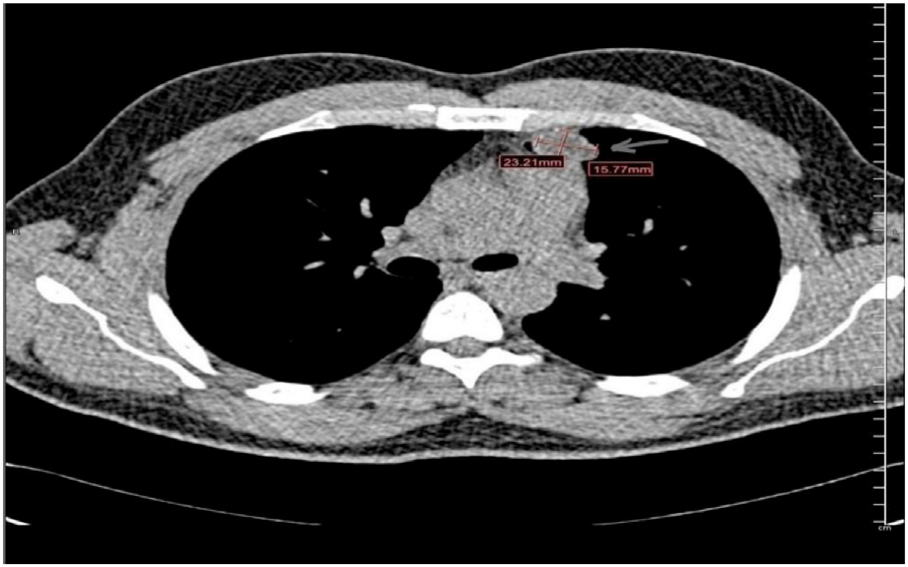
CT scan of the chest with contrast, demonstrating an anterior mediastinal hypodense mass 23.2 × 15.7 mm in size.

**Fig. 2:**
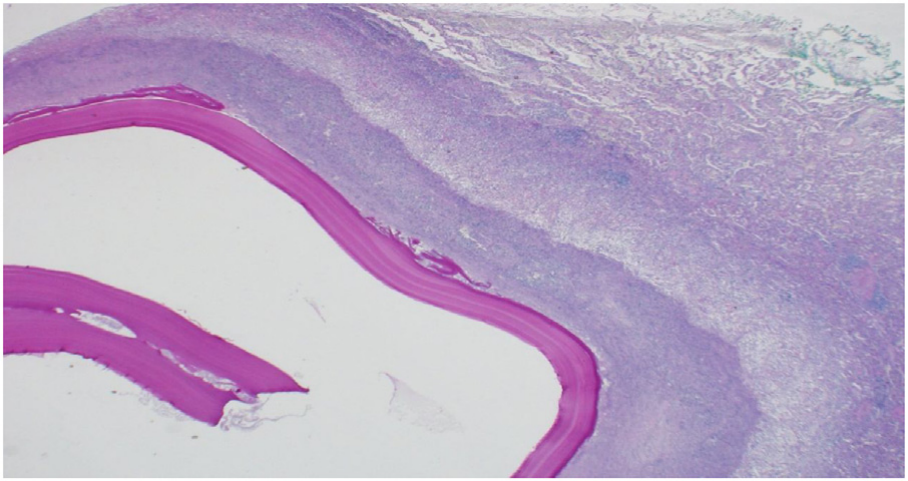
Light microscopic view showing laminated membrane at the center surrounded by necrotizing granulomatous inflammation.

A 36-year-old gentleman, active smoker with no history of chronic medical illnesses, presented in June 2020 with chief complaint of left sided chest pain for two weeks. The pain was moderate in intensity, sharp in nature and increased with inspiration with no radiation. He did not have any history of dyspnea, cough, tachypnea, fever, night sweats, or weight loss. There was no history of trauma and review of other systems was negative. History was negative for recent exposure to pets or farm animals. Physical examination and vital signs were essentially normal. Lab results were unrevealing except for positive *Echinococcus granulosus* antibodies in the serum (hemagglutination and latex agglutination tests). Computed tomography (CT) scan of the chest and abdomen revealed an anterior mediastinal hypodense mass 23.2 × 15.7 mm in size (Fig. 1). No lesions were detected in the pulmonary or hepatic parenchyma. This was followed by CT guided aspiration biopsy which showed necrotic material with laminated membrane highly suggestive of hydatid cyst (Fig. 2).

The patient received oral albendazole for two weeks and subsequently underwent removal of the hydatid cyst by VATS. Pathological analysis of the resected mass was also consistent with hydatid cyst. The patient had an uneventful course following surgery and was discharged home four days later. He was seen in thoracic surgery and infectious disease clinics at one-week post-discharge and was feeling very well with no reportable symptoms. Repeat Posteroanterior and lateral chest X-rays two weeks after the surgery showed post-operative changes in the mediastinum with no masses, pleural effusion or pulmonary consolidation.

## Discussion

After entering the intestine, *E. granulosis* gains access to the liver and lungs through systemic circulation. Usually, these organs act as barriers against further spread which explains the rarity of additional organ involvement [3]. Depending on the site of Intrathoracic extrapulmonary hydatidosis and compression of nearby structures, patients can present with a variety of symptoms, including chest discomfort, shortness of breath, dysphagia, and dysphonia [4]. The incidence of mediastinal hydatidosis constitutes 0.1 % of all cases of hydatidosis and can present with a wide range of clinical pictures. Anterior mediastinal cysts were detected in patients who had significant airway compromise, middle cysts in patients who had compression of the major intrathoracic vessels, and posterior cysts in patients who presented with paraplegia due to intravertebral extension and spinal cord compression. Histopathology and serologic testing confirmed the diagnosis of hydatidosis. Depending on the complexity of the location, different surgical approaches and techniques were deployed including thoracotomy and laminectomy. The majority of the patients had a satisfactory post-operative course [5]. Isolated diaphragmatic involvement was reported before in a young female who presented with non-specific right hypochondrial pain for two months duration. Further work up revealed a diaphragmatic cyst which was resected through thoracotomy. Subsequent histopathological analysis confirmed the diagnosis of hydatidosis. She received postoperative albendazole and was discharged shortly after the procedure. She had an excellent outcome with no recurrence detected at three months follow up [6].

Multiple mechanisms were proposed for secondary mediastinal involvement and include fissuring from the lung and trans-diaphragmatic and lymphatic spread from the abdomen [7]. However, the underlying mechanism by which primary mediastinal hydatidosis occurs, remains a debatable subject. Among fourteen cases of mediastinal hydatidosis, only six patients had isolated mediastinal cysts, with the other cases having hepatic and pleural involvement. Most of the cysts were located in the anterior mediastinum [8].

Surgical treatment is of utmost importance in such cases, as the role of medical treatment alone with albendazole has not yet been fully established. Surgical modalities successfully deployed before include sternotomy, thoracotomy and VATS [9]. Our patient underwent successful resection of the cyst with VATS preceded by two weeks of oral albendazole. He remained symptom-free at follow up clinic visit three months post discharge. Most likely, he acquired the infection after he had eaten raw, unwashed vegetables.

## Author contribution statement

Almurtada Razok performed literature review, formal analysis, and wrote the original draft and review of the manuscript. Maisa Ali analyzed the case and wrote the editing and review of the manuscript. All authors approved the final version for submission.

## Funding statement

This article did not receive any specific grant from funding agencies in the public, commercial, or not-for-profit sectors.

## Consent

Written informed consent was obtained from the patient for publication of this case report. A copy of the written consent is available for review by the Editor-in-Chief of this journal on request.

## CRediT authorship contribution statement

**Almurtada Razok:** Writing - original draft, Writing - review & editing, Formal analysis. **Maisa Mohamed Bashir Ali:** Writing - review & editing.

## Declaration of Competing Interest

The authors report no declarations of interest.

## References

[1] Zhang W., Li J., McManus D.P. (2003). Concepts in immunology and diagnosis of hydatid disease. Clin Microbiol Rev.

[2] Varshney M., Shahid M., Maheshwari V., Siddiqui M.A., Alam K., Mubeen A. (2011). Hydatid cyst in tail of pancreas. BMJ Case Rep.

[3] Gasmi M., Fitouri F., Sahli S., Sghairoun N., Hamzaoui M. (2010). Hydatidose médiastinale primitive chez l’enfant: à propos de deux cas. Rev Pneumol Clin.

[4] Msougar Y., Afandi O., Ihfa N., Baiz Y., Rouiessi Y., Khellouki M. (2013). Mediastinal hydatid cyst: a case report. J Med Case Rep.

[5] Rakower J., Milwidsky H. (1960). Primary mediastinal echinococcosis. Am J Med.

[6] Salih A., Kakamad F., Rauf G. (2016). Isolated hydatid cyst of the diaphragm, a case report. Int J Surg Case Rep.

[7] Eroglu A., Kurkcuoglu C., Karaoglanoglu N., Tekinbas C., Kaynar H., Onbas O. (2002). Primary hydatid cysts of the mediastinum. Eur J Cardiothorac Surg.

[8] Zidi A., Zannad-Hantous S., Mestiri I., Ghrairi H., Baccouche I., Djilani H. (2006). Kyste hydatique primitif du médiastin: 14 cas [Hydatid cyst of the mediastinum: 14 case reports]. J Radiol.

[9] Eroğlu A., Aydın Y., Altuntaş B., Ulaş A.B. (2016). Surgical management of primary mediastinal hydatid cysts: a 30-year experience. Turk Gogus Kalp Dama.

